# Knowledge, practices and barriers to access sexual health of women in the menopausal stages: a cross-sectional study with Brazilian gynecologists

**DOI:** 10.1186/s12905-024-02901-x

**Published:** 2024-01-18

**Authors:** Amanda Oliveira de Carvalho, Lucas Barrozo de Andrade, Flávia Fairbanks L. O. Ruano, Cristina Maria Duarte Wigg, Lizanka Paola Figueiredo Marinheiro

**Affiliations:** 1 Instituto Nacional de Saúde da Mulher, da Criança e do Adolescente Fernandes Figueira - Fundação Oswaldo Cruz (IFF/Fiocruz), Avenida Rui Barbosa, 716, Flamengo, Rio de Janeiro, RJ CEP 22250-020 Brazil; 2 Universidade Federal do Rio de Janeiro, Rio de Janeiro, RJ Brasil; 3 Universidade de São Paulo, São Paulo, SP Brasil; 4 Miller School of Medicine - University of Miami, Miami, FL USA

**Keywords:** Sexual health, Menopause, Gynecology, Health knowledge, Attitudes, Practice, Surveys and questionnaires

## Abstract

**Background:**

Sexual health access and care for women in the menopausal stages face significant barriers, presenting deficits in relation to diagnosis and treatment. Although epidemiological data indicate high prevalence of problems related to sexual health in this population, traditionally, the theme is not discussed in health care settings. This study aimed to analyze knowledge, practices and barriers to access sexual health of women in the menopausal stages in the context of women’s health care in Brazil.

**Methods:**

With a cross-sectional design, a questionnaire was distributed electronically, encompassing variables related to knowledge; practices; and barriers to access sexual health of women in the menopausal stages. The data obtained were subjected to analysis using both descriptive and inferential statistics. Specifically, we employed multivariate analysis, employing multiple linear regression models, to discern potential factors associated with outcomes concerning the level of knowledge and the frequency of addressing the topic in professional practice.

**Results:**

The sample included 70 physicians with specialization in obstetrician/gynecologists who work in health care with women in the menopausal transition or postmenopausal women. A high level of self-reported knowledge about sexual health was identified. Regarding the practices, most of them reported directly proposing the subject and not using instruments. Although they reported frequently addressing the topic in general, topics related to vaginal lubrication, dyspareunia, and sexual dysfunction have been more present in the clinic compared to sexual orientation and women’s relationship with themselves. The main barriers were time limitation and patient discomfort with the topic. The multivariate models indicated that female gynecologists and professionals with higher levels of knowledge on the subject had a higher frequency of addressing sexual health in clinical practice with women in menopausal stages.

**Conclusions:**

Sexual health access and care for brazilian women in the menopausal stages presents discrepancies in the frequency of approach between the various topics, in addition to the predictive character of technical knowledge in the practices of professionals. To ensure universal access to sexual health services for this population, an active approach through specific instruments is important, as well as the reinforcement of strategies to improve the level of knowledge of professionals.

**Supplementary Information:**

The online version contains supplementary material available at 10.1186/s12905-024-02901-x.

## Background

Sexual health access and care for women in the menopausal stages face significant barriers [1, 2], presenting deficits in relation to diagnosis and treatment [1–4]. Although epidemiological data indicate high prevalence of problems related to sexual health [5], suggesting an expressive impact on public health, the understanding of sexuality is marked by prejudices and taboos, so that, traditionally, the theme is not discussed in health care settings [3, 6]. In the study by Evcili and Demirel [7], although many health professionals recognized that the evaluation of sexuality is part of an integral care, not all of them performed it. A similar result to that of Bdair and Maribbay [8], who identified that about 70% of the professionals evaluated recognize the importance of to access sexual health, but rarely do it with the patients. Other studies pointed out that some professionals have doubts if sexual health is part of their scope of practice and which care professional is responsible for addressing issues related to sexuality [9].

Communication about sexuality in the clinical setting is still particularly important considering that the demand may not be presented spontaneously by women. In the study by Wong et al. [10], only 1.4% of the sample had sought health care reporting some sexual complaint; however, after the evaluations, high prevalence of sexual problems and low quality of sexual life were revealed, especially in postmenopausal women. In an extensive study on the sexual behavior of Brazilians, Abdo et al. [11] suggested that there is underreporting of sexual complaints to health professionals. Considering that addressing sexual health among women in the menopausal and menopause-related stages is an important tool for the diagnosis of sexual health issues [1] and other issues that affect women’s lives [12], health professionals who have contact with women’s sexual complaints should not neglect the subject, but approach it in a carefully way [13].

Thus, gynecologists should access sexual health issues during routine gynecological appointments [14]. However, these professionals have been aware of less than 50% of patients’ sexual problems [15]. In the Brazilian context, research on the approach to sexuality in health care pointed out that gynecologists were not being adequately prepared to address sexual complaints [16]. In a Brazilian study on obstetrician/gynecologists (ob/gyns) approach to sexuality during pregnancy, Vieira et al. [17] pointed out that the training does not adequately prepare professionals for demands related to sexuality. Another Brazilian study indicated that most ob/gyns had received no or little training on this topic [18]. Thus, many sexuality issues are not often addressed in gynecological care [4, 19].

It is important, therefore, to understand the current Brazilian panorama regarding the access to sexual health, specifically with women in the menopausal stages, in order to identify demands in the field and develop appropriate strategies to enable health care attentive to the specific experiences of women in this period of life. Thus, this study aimed to analyze the knowledge, practices and barriers perceived by obstetrician/gynecologists regarding the access sexual health of women in the stages of menopause, also verifying possible factors associated with the knowledge and practices.

## Methods

### Design and setting of the study

The study, employing a cross-sectional design, was conducted in a digital environment, taking into consideration the timeframe between 2020 and 2022, during which Brazil and the world were grappling with the COVID-19 pandemic and implementing social distancing measures as a preventive strategy against contagion. As a result, data collection was carried out through digital means, utilizing a self-report questionnaire (websurvey) between November 2021 and May 2022, marking the development period of this study.

### Participants

The sample was composed of 70 professionals with medical training and specialization in obstetrician/gynecologists recognized in Brazil (inclusion criteria). The exclusion criteria was not working in health care with women in the menopausal transition or postmenopausal. The measurement of this variable was carried out through the participant’s self-report and no specific analysis was performed between the different phases of menopause. The participants were recruited through the social networks Instagram, Whatsapp and Facebook; in addition to official communication spaces of the category, through the collaboration of the ob/gyns associations of the following Brazilian states: São Paulo, Mato Grosso do Sul, Pernambuco and Paraná. Data collection on social networks was carried out for 6 months, between November 2021 and May 2022. Contact with Gynecology Associations happened between January and April 2022. The sampling method employed in this study was convenience sampling, a type of non-probabilistic sampling. As a consequence, the findings of this research cannot be extrapolated or generalized to other populations.

### Measures

Data were collected using a questionnaire developed by the study team based on scientific literature in the area. The average time taken to complete all the survey was 15 min. Systematized bibliographic research made it possible to survey the topics and issues pertinent to the theme and, therefore, allowed the identification and selection, with scientific rigor, of a set of variables to be investigated in the study. In general, the questionnaire was inspired and adapted from the data collection tool developed by Sobecki et al. [4] for a survey with gynecologists/obstetricians in the United States of America on the approach to sexuality in health care.

The set of variables identified in the scientific literature was grouped into three thematic dimensions: Knowledge; Practices; and Barriers. The Knowledge dimension aimed to assess self-perception about technical/theoretical knowledge; sense of responsibility; importance; and relevance of the theme in health care. The items were organized in affirmative format, with response options ranging from strongly agree to strongly disagree. The Practices dimension was divided into items about the sexual health approach method and sexual health approach technique; in addition to the frequency of addressing general and specific topics related to sexual health, with response options ranging from very often to never. The Barriers referred to participants’ perceptions of possible challenges to access sexual health in women’s health care throughout reproductive aging. In this last section, in a list format, one or more items could be marked in a yes/no format.

Besides these thematic dimensions, the questionnaire included a section on the socioeconomic and professional characterization, in order to identify the profile of the study participants. The following variables were included: biological sex; gender identity; race/color [20]; age; income (Per Capita Household Income) [21]; time of medical degree; time of specialization in ob/gyns; training/specialization in sexuality; age profile of patients; state of work; health care system of work; level of care; and health care modality.

Considering that the content validity of the instruments depends on the elaboration procedures adopted, the development of the questionnaire encompassed the identification of the dimensions of interest in the scientific literature; construction of the items; and consultation with experts [22]. Among the specialists, we included professionals with extensive knowledge and clinical and methodological experience in the field. Regarding reliability, the analysis through Crombach’s Alpha indicated that the knowledge and practice dimensions presented reliable results and measured the constructs accurately (α = 0.83 and α = 0.87, respectively). With respect to the barriers, this analysis was not performed since the sum of the items did not refer to a single category. In this case, the data for each item were coded as dichotomous yes/no categorical variables.

### Data analysis

Considering the research objectives, the following outcomes to be analyzed in the study were defined: level of knowledge regarding the theme (Knowledge dimension); and frequency of access to sexual health topics in clinical practice with women in the menopausal stages (Practice dimension). Thus, to perform analyses by dimension, the answers in each item were numerically coded and a total variable relative to the sum of the items of the Knowledge dimension and another total variable relative to the Practice dimension items were elaborated.

The variables in this study were classified based on the nature of their measurement as either quantitative or qualitative. In the descriptive analyses, qualitative variables were encoded as factors and examined in terms of relative and absolute frequencies. On the other hand, quantitative variables were coded as numerical and their values were presented using the mean and standard deviation (SD).

In order to explore the effect of sociodemographic and professional variables on the level of knowledge and frequency of access in the practice of obstetrician/gynecologists professionals, multivariate linear regression models were performed for each dimension (Knowledge and Practice). To select the variables of interest to integrate the regression models, the conceptual model of each outcome and the collinearity analysis by Variance Inflation Factor (VIF) were considered. Thus, in the multivariable regression model for the analysis of factors associated with Knowledge, the variables sex; race/color; age; household income; training/specialization in sexuality; age profile of patients; and health care modality were included. As for the regression model for Practices, the following sociodemographic and professional variables were included: sex; race/color; age; training/specialization in sexuality; household income; age profile of patients; health care modality; knowledge level; sexual health approach method; and sexual health approach technique. No regression model was performed for the Barriers section considering that the sum of the items did not refer to a single dimension.

The analyses were performed in Software R and Rstudio (version 4.1), using the Tidyverse, Psyco, SummaryTools, Janitor, and DataExplorer packages. A 95% confidence interval and elected significance level of 5% (α = 0.05) was used.

## Results

The study sample consisted of 70 participants. Most were female (80%), self-declared white (71.40%) and, in terms of gender identity, stated themselves as “woman” (23.44%) or “female” (48.44%). The age of the sample ranged from 29 to 83 years, with an average of 47 years of age, 22 years of medical degree, and 18 years of specialization in ob/gyns. Most had no training/specialization in sexuality (81.40%) and worked in clinical practice mainly with women between 40 and 49 years old (41.20%). The sample was composed of professionals from 17 Brazilian states, 50% from the state of São Paulo and 21.43% from Rio de Janeiro. Most of them worked in the private health system (90%); and/or in the public health system - municipal level (45.70%). Most said they worked in secondary care (63.80%) and with an exclusively face-to-face health care (55.70%) (Table 1).

**Table 1:** Sociodemographic and professional characteristics of the study participants

Variable (*N* = 70)	N / Average	% / DP
**Biological Sex**		
Female	56	80.00%
Male	14	20.00%
**Gender Identity**		
Female	31	48.44%
Woman	15	23.44%
Male	9	14.06%
Man	2	3.12%
Other	7	10.94%
**Race/Color**		
White	50	71.40%
Black	17	24.30%
Other	3	4.30%
**Age (years)**	47.2	13.1
(29–83)		
**Household income (R$)**	15.365.07	13.752,48
(2000–80,000)		
**Time of medical degree (years)**	22.0	12.6
(3–55)		
**Time of specialization in ob/gyns (years)**	18.6	12.7
(1–53)		
**Training/Specialization in sexuality**		
No	57	81.40%
Yes	13	18.60%
**Age profile of patients (years)**		
Under 30	6	8.80%
From 30 to 39	24	35.30%
From 40 to 49	28	41.20%
50 to 59	9	13.20%
60 and over	1	1.50%
**Health Care Modality**		
Face-to-face health care only	39	55.70%
Face-to-face health care and Telemedicine	31	44.30%
**State**		
São Paulo	35	50%
Rio de Janeiro	15	21.43%
Others	20	28.57%
**Health Care System**		
Private Health System	63	90.00%
Public Health System - municipal level	32	45.70%
Public Health System - state level	16	22.90%
Public Health System - federal level	11	15.70%
**Level of Care**		
Primary Care	40	58.00%
Secondary Care	44	63.80%
Tertiary Care and Hospitalization	42	60.90%
**Sexual Health Approach Method**		
Passive Approach	18	25.70%
Active Approach	52	74.30%
**Sexual Health Approach Technique**		
Not using instruments	52	74.30%
Using instruments	18	25.70%

In the Knowledge dimension, it was verified a high level of self-reported general knowledge by the sample regarding the theme. The highest averages were observed in items related to the sense of importance of the theme in Health Care, with an average of 4.91 (SD = 0.33); its inclusion as part of the routine care to this population (Mean = 4.84, SD = 0.56); and sense of responsibility of this professional body towards the theme (Mean = 4.78, SD = 0.57). The lowest mean corresponded to the item related to the self-perception of technical and theoretical knowledge regarding the approach to sexual health, specifically with women in the menopause-related phases with mean of 4.10 (SD = 0.90), followed by the self-perception of knowledge regarding the approach to sexual health in general (Mean = 4.16, SD = 0.85).

Regarding Practices, when questioned in general, most of the sample reported addressing patients’ sexuality during health care very often (48.57%) or frequently (41.43%). In specific terms, the most frequently approached variables, on average, were vaginal lubrication (Mean = 4.66, SD = 0.61); dyspareunia (Mean = 4.60, SD = 0.52); and sexual dysfunctions (Mean = 4.51, SD = 0.63); while the topics least addressed by professionals were sexual orientation, with mean 3.60 (SD = 1.10) and relationship with oneself (Mean = 3.59, SD = 1.06); followed by arousal (Mean = 3.91, SD = 0.96) and orgasm (Mean = 3.81, SD = 1.03). Regarding the method of approaching, the majority (74.30%) reported proposing the topic directly (active approach to sexual health) and, in terms of technique, the minority used instrument/questionnaire/interview model for this (25.70%).

**Fig. 1:**
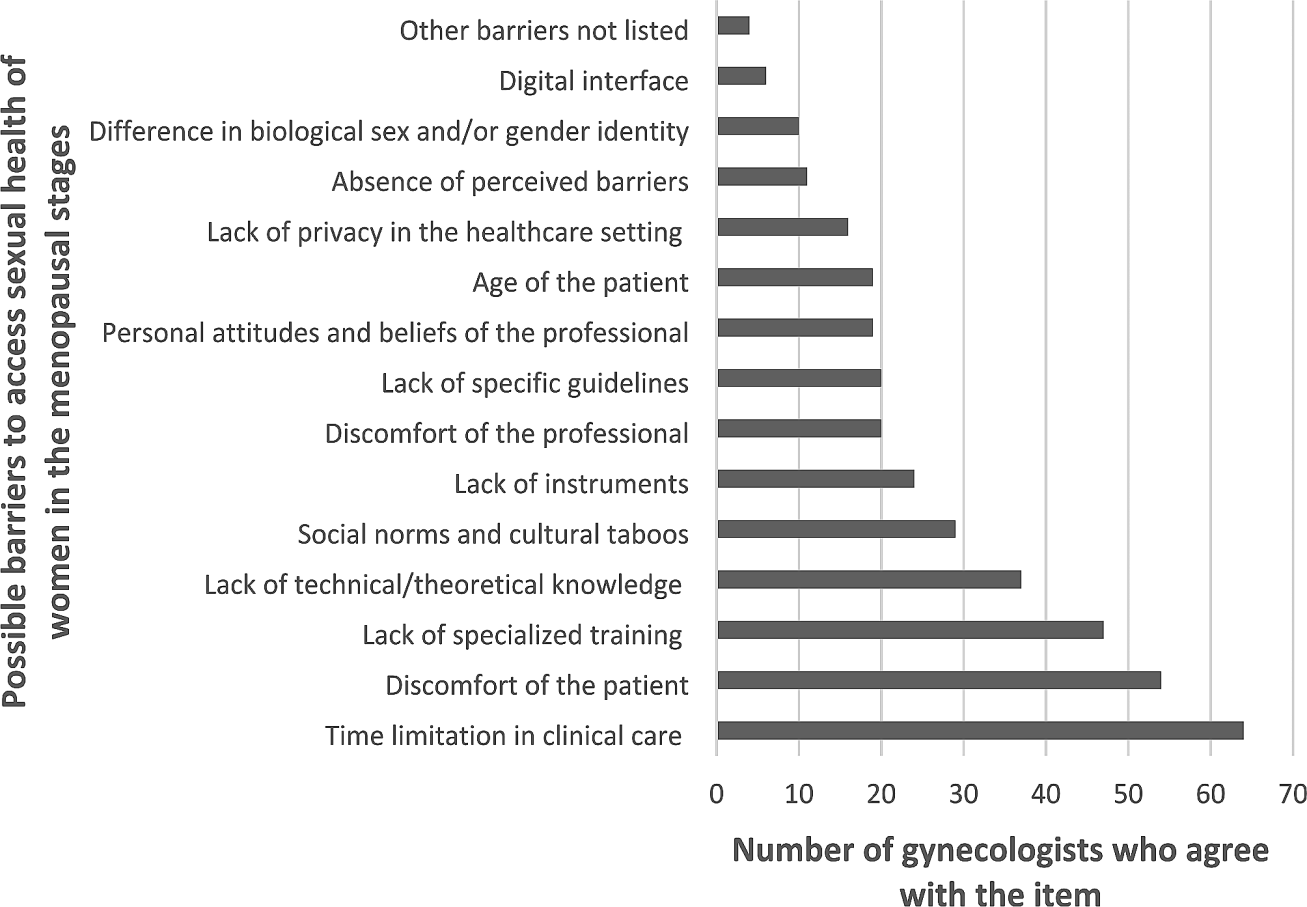
Barriers perceived by participants to approach sexual health in health care with women in the menopausal stages

Regarding the Barriers, as shown in Figs. 1, 64% of the professionals pointed out the limitation of time in setting care as a barrier to address sexual health issues. Other main barriers pointed out by the professionals were the patient’s discomfort with the theme (54%); lack of specialized training (47%); lack of technical/theoretical knowledge (37%); social norms and cultural taboos (29%); and lack of specific instruments (24%).

The relationship between the level of knowledge regarding the approach to sexual health and the sociodemographic and professional characteristics is in Table 2. No significant differences were found in the level of knowledge from the independent variables analyzed, although the variables specific training/specialization in sexuality and modality of care presented marginally significant results (*p* = 0.09 and 0.099, respectively). The explanation power of this model was approximately 19% (R^2^ = 0.1876, *p* = 0.53).

**Table 2:** Relationship between the level of knowledge regarding the approach to sexual health and sociodemographic and professional characteristics

Variable	SS	DF	MS	F	P valor	ηp2	CI 90%
Intercept	239.33	1	239.33	31.87	< 0.001		
Sex	13.28	1	13.28	1.77	0.19	0.04	[0.00, 0.15]
Race/Color	11.63	3	3.88	0.52	0.673	0.03	[0.00, 0.09]
Age	10.31	1	10.31	1.37	0.247	0.03	[0.00, 0.14]
Household income	0.02	1	0.02	0	0.956	0	[0.00,1.00]
Training/specialization in sexuality	22.46	1	22.46	2.99	0.09	0.06	[0.00, 0.19]
Age profile of patients	13.83	4	3.46	0.46	0.764	0.04	[0.00, 0.08]
Health care modality	21.26	1	21.26	2.83	0.099	0.06	[0.00, 0.18]

F(12, 49) = 0.94, p valor = 0.52

R2 = 0.1869

**Fig. 2:**
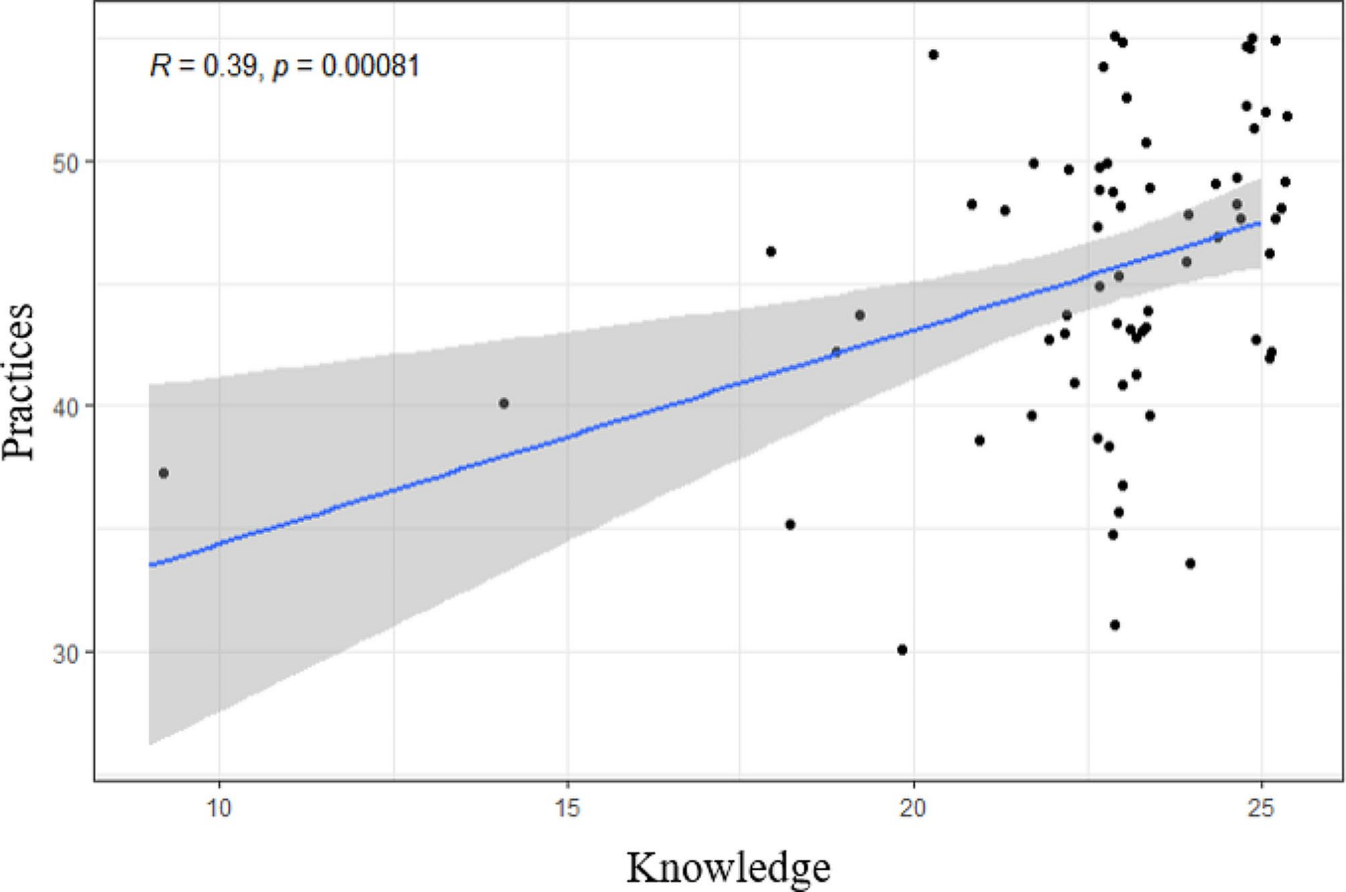
Correlation between level of knowledge and frequency of access sexual health in clinical practice with women in the menopausal stages

The analysis of the factors associated with variability in the frequency of access sexual health topics in clinical practice with women in the menopausal stages (Table 3) identified that gender and level of knowledge of professionals explained a statistically significant change. In this case, female gynecologists showed a significantly higher level in the frequency of access when compared to males (Δ = 5.35, SE = 2.17, *p* = 0.018). In relation to the level of knowledge, a proportionally significant relationship was verified (Fig. 2). In general, the results pointed out that every 1 point more that the person presents on the knowledge scale, on average, he or she increases 0.298 points on the practices scale (β = 0.298, SE = 0.012, *p* = 0.012). As for the model fit, the explanatory power of these variables was 53%, with a significant predictive value (R^2^ = 0.53, *p* < 0.001).

**Table 3:** Relationship between frequency of access sexual health in clinical practice, sociodemographic and professional characteristics, and level of knowledge

Variable	SS	DF	MS	F	P valor		ηp2		CI 90%	
Intercept	18.58	1	18.58	0.75	0.39					
Biological Sex	149.84	1	149.84	6.08	0.018*		0.12		[0.01, 0.27]	
Race/Color	127.93	3	42.64	1.73	0.174		0.1		[0.00, 0.21]	
Age	41.07	1	41.07	1.67	0.203		0.04		[0.00, 0.16]	
Training/specialization in sexuality	17.83	1	17.83	0.72	0.4		0.02		[0.00, 0.12]	
Household income	9.56	1	9.56	0.39	0.536		0.01		[0.00, 0.10]	
Age profile of patients	78.04	4	19.51	0.79	0.537		0.07		[0.00, 0.13]	
Health care modality	14.45	1	14.45	0.59	0.448		0.01		[0.00, 0.11]	
Knowledge level	169.43	1	169.43	6.87	0.012*		0.13		[0.02, 0.28]	
Sexual health approach method	29.19	1	29.19	1.18	0.282		0.03		[0.00, 0.14]	
Sexual health approach technique	89.12	1	89.12	3.62	0.064		0.07		[0.00, 0.21]	

F(15, 45) = 3.42, p valor < 0.001

R2 = 0.53

*p < 0.05

Although multivariate analysis indicated no significant association between frequency and sexual health approach form and technique, bivariate analyses did alert associations. Thus, possibly in conjunction with other variables, the predictive ability of these variables was further explained by the variance of other factors. The result of the Practices dimension, regarding the frequency of access specific topics, was significantly higher in the group of professionals who actively access the topic than in the group of professionals who reported waiting for the patient to show interest (t(34) = 3.265, *p* < 0.01, d cohen = 0.84). The effect measurement evidenced that the data, besides being significant, present a difference of practical relevance. Regarding technique, professionals who use instruments in clinical practice showed a significantly higher frequency of addressing topics related to sexual health than those who do not use them (t(33,24) = 2.2, *p* = 0.035, d cohen = 0.57).

## Discussion

This study described the knowledge, practices and barriers perceived by Obstetrician/gynecologists professionals from several states in Brazil regarding the approach to sexual health of women in the menopausal stages, also verifying factors associated to knowledge and practices. The results indicated a high level of self-reported knowledge by ob/gyns professionals regarding the perception of technical skills; responsibility; relevance and importance of the theme. Regarding practices, although they reported frequently addressing the topic in general, there were discrepancies regarding the frequency of addressing specific topics. The professionals who approach the theme actively and those who use instruments showed a significantly higher frequency in approaching topics related to sexual health. Furthermore, gender and knowledge level of the professionals acted as predictors for the frequency of addressing these topics. Specifically, female gynecologists and professionals with higher levels of knowledge had a higher frequency of addressing sexual health in clinical practice with women in the menopausal stages. Regarding the barriers perceived by professionals, the main ones were time limitation and discomfort of patients, followed by lack of specialized training, technical/theoretical knowledge, social norms and cultural taboos, and lack of specific instruments.

The analyses related to the knowledge of ob/gyns professionals indicated high agreement of the sample with the importance of the topic; its inclusion in the health care routine with women in the stages of menopause; and the recognition of the responsibility of this professional body facing the topic. These results corroborate the findings of Rabathaly and Chattu [23] who identified that 96% of the professionals believed that sexual function is important in this stage of life; while 11% believed that sexual health care has little relevance for the well-being of this population and 29% of the professionals believed that it is not a priority. Recognizing the relevance and importance of the topic to the health care of this population are urgent considering the current prevalence of sexual complaints and problems [5]. Furthermore, many of these issues can be resolved if the ob/gyns provider addresses this topic and provides patients with information about sexuality and the various aspects of sexual response [15]. Reinforcing, thus, the responsibility of this professional body facing the theme, considering also that most women who receive gynecological care report concerns about sexual health [24] and that this is an area of medical practice allowed to the specialists in ob/gyns [25].

Furthermore, basic knowledge about sexual response and biopsychosocial aspects of the construction of sexuality is part of the competencies to be developed in the training of this specialist [26]. In this sense, the professionals indicated high levels of knowledge. However, in comparison to the other items evaluated in terms of knowledge, the lowest results were regarding the self-perception of technical and theoretical knowledge, in general and specifically with this public. Vieira et al. [17] in a study about the ob/gyns approach to sexuality during pregnancy, pointed out that many professionals felt unprepared because of deficiencies in their training regarding the area of human sexuality in general and specifically during pregnancy. In the study by Rabathaly and Chattu [23], 14% of the professionals did not feel able to approach sexuality issues with older patients. However, considering that menopause is one of the most significant events in women’s lives, it is important that adequate care be offered in health services [27]. To this end, it is necessary that the training of these professionals address the health demands of the population, so that the gap between advances in sexual medicine and the clinical skills of professionals can be overcome [28]. For a productive dialogue about sexuality, it is necessary that the professional feels prepared and comfortable to discuss this theme [17].

From this perspective, the results of the multivariate analysis performed in this study corroborated the importance of professional training to prepare these specialists, in terms of technical and theoretical knowledge, to deal with the theme in clinical practice since the level of knowledge of professionals acted as a predictor for the frequency of addressing specific topics related to sexual health. Thus, indicating that the higher the level of knowledge on the subject, the greater the frequency of addressing sexual health in clinical practice with women in menopausal stages. This relation had already been proposed in another Brazilian study with ob/gyns professionals on the theme, carried out in 1986, which indicated that this professional, despite having a high level of contact with the patients’ sexuality, had not been adequately prepared to attend to these complaints [16]. In this study, Rodrigues Junior and Costa [16] proposed that the lack of training and professional preparation interfered in the care regarding sexuality. In another Brazilian study with ob/gyns professionals on the topic, in 2015, Vieira and collaborators reinforced that most did not feel competent or safe to deal with the topic and attributed this difficulty to lack of knowledge [18]. In a survey of primary care physicians from Trinidad and Tobago, professionals with higher knowledge scores were three times more likely to talk about sexuality with patients over 45 years old [23].

The results of this study also indicated the perception of the professionals themselves about the importance of technical preparation for clinical practice, since they reported the lack of specialized training and lack of technical/theoretical knowledge as one of the main barriers. These findings are in line with the literature in the area. Sobecki et al. [4]in a study with ob/gyns professionals in the USA, identified that the improvement in the practices of these professionals regarding communication on sexual issues in women’s health depends, among other issues, on training and continuing education. Medical students evaluated in a study in Mexico reported that they feel unable to provide sexual health solutions because their training is insufficient to respond to the care needs of the population [29]. In this sense, Parish and Clayton [28] had pointed out that medical students, residents, and practitioners received variable, nonstandard, and inadequate training on sexuality communication, assessment, and treatment. Specific to ob/gyns training, Lara and colleagues [15] pointed out that most residents are ill-equipped to deal with sexual problems. Consequently, health care providers have gaps in knowledge about assessment and treatment of sexual problems [30] and, as a result, feel uncomfortable in addressing the topic [31]. Therefore, sexual health education needs to be improved in the training of health professionals [32]. Therefore, sexual health education needs to be improved in the training of health professionals, considering that it could minimize this embarrassment and contribute to qualify the performance of these professionals, aiming to make them familiar even with the different concepts of gender, sexual orientation, and identity [33].

Thus, professionals will be better able to address these specific topics, which, in view of the results of this study, have been neglected in Health Care with women in the stages of menopause. The professionals evaluated reported addressing more frequently vaginal lubrication and sexual dysfunctions compared to topics related to sexual orientation and the woman’s relationship with herself. This result is similar to the findings of Rabathaly and Chattu [23] who reported that less than 50% of providers approached sexual orientation in primary care; as well as in research with ob/gyns in the US, who tended not to talk about sexual identity and orientation with patients [4]. Thus, reinforcing the difficulties experienced in accessing and providing health care for LGBTI (Lesbian, Gay, Bisexual, Transvestite, Transgender, and Intersex) populations and other non-normative bodies and identities [34]. Aspects related to arousal and orgasm also showed lower frequencies, as in the study by Sobecki et al. [4]. Despite the importance of these topics for the diagnosis and/or treatment of many gynecological conditions. Sexual health assessment should therefore be based on a thorough history, including sexual activity and function; sexual and gender identity; sexual orientation; relationship and partnership; general health and comorbidities; as well as cultural and personal expectations and attitudes [35].

In other studies on addressing sexual health in health care, the difference in sex or gender identity between patient and professional has been identified as a barrier to dialogue about sexuality [4, 23, 36]. In the present research, only 10% of the sample identified this issue as a barrier, however, the variable sex was a statistically significant predictor for the frequency of addressing topics related to sexual health. Thus, indicating that female gynecologists reported addressing this topic more frequently. This result is similar to that of Sobecki et al. [4] where female gynecologists were the most likely to ask patients about sexual activity and sexual orientation. In this sense, Rabathaly and Chattu [23] pointed out that gender and age agreement was a predictor for increasing practitioners’ comfort in discussing sexual health with patients. Similarly, in a survey of graduate students in Psychiatry, age and gender difference between professional-patient were among the difficulties indicated when dealing with the topic of sexuality in mental health assessment [36]. In this study, 19% indicated the patient’s age as a possible barrier. It is worth mentioning, from this point of view, that the sample analyzed was composed mostly by women; who attend the public between 40 and 49 years old; and with a mean age of 47 years - within the age range that menopause occurs for most Brazilian women [37]. Representing, therefore, a sample, in general, in line with the population of the study questions - women in the stages of menopause.

Regarding the form of approach, professionals who said they approached the topic directly (active approach), presented significantly higher frequency of addressing topics related to sexual health than professionals who wait for the demand to be presented by the patient (passive approach). The latter, therefore, may present disadvantages regarding health care, since many women do not present their sexual complaints unless they are given an opening by the professional. In a study with Chinese women of different stages of menopause, only 1.4% had sought specialized help for sexual problems, despite having presented a high prevalence of sexual dysfunctions when evaluated [10]. The results of Cuerva et al. [1] indicated, in this sense, an increase of 35.9% in the prevalence of sexual commitment after the active approach of professionals on the subject, so that most cases of sexual dysfunction would not have been detected if the theme was not proposed by the professional. Thus, despite the high prevalence, it is unlikely that women present their sexual demands without being asked [31]. This scarcity of spontaneous reports indicates, therefore, the importance of professionals approaching the issue in an active way [38, 39]. This scarcity of spontaneous reports, therefore, indicates the importance of professionals actively addressing these issues, opening a door for patients to know that they have been offered a free area for these issues [40]. However, this is not yet the reality in all health services. In this research, 25.70% reported addressing the theme only after the demand is presented by the patient. Other studies have pointed to even more alarming data: Rabathally and Chattu [23] indicated that 99% of the professionals approached sexual health after the complaint was raised by the patient; while in Seitz and collaborators [41] 25% of the physicians suspected sexual health problems in their patients, but did not ask about it. According to Vieira et al. [17] there is a silent conspiracy in which neither the patient nor the professional introduces the subject.

In a broad survey on the sexual profile of the Brazilian population, Abdo and collaborators [11] identified that many women do not feel comfortable talking about sex, being more inhibited in this sense than men. This embarrassment has profound implications for research, prevention and treatment in the field of sexual health [11]. Most professionals seem to recognize the difficulty women have in introducing the topic during a consultation [42]. Many even report that this is a barrier to addressing the topic in health care. In research with mental health professionals, Seitz et al. [41] identified that the embarrassment of the patient was among the reasons mentioned as barriers, while in the study by Bungener et al. [43] 31.4% of professionals reported not discussing the topic because patients would feel uncomfortable/ashamed and 20.2% reported not approaching it because they themselves were embarrassed. In this survey, 54% of ob/gyns providers indicated the patient’s discomfort as a barrier and 20% the provider’s own discomfort. With this context, many sexual health problems are not diagnosed and treated because of patient or professional inhibition [19] since both may consider it embarrassing to discuss issues related to sexuality [30].

This shyness and discomfort in talking about sex may be a reflection of the rigidity of cultural, religious, and moral values that permeate the experience of sexuality [44, 45]. In this sense, 29% of the professionals indicated that social norms and cultural taboos related to the theme act as barriers to approach sexual health with the patients; while 19% indicated, among the obstacles, attitudes and personal beliefs of the professional. Sexuality, as an activity related to cultural and social values [5] is the target of moral, religious or scientific norms that are learned since childhood [46]. In many contexts, even, sexuality is seen as a private matter, with no room to be addressed in health care settings [47]. It is emphasized, however, that although the approach to sexual health needs to be judicious, considering the most intimate aspects of the theme, it should not be neglected [13].

With regard to technique, on the other hand, most professionals reported that they do not use interview instruments or models; although their use has significantly increased the frequency of approach. Thus, in agreement with Abdo [13], the FSD data in Brazil justify the need for a routine investigation of sexual health and the development of instruments that facilitate this process. In this sense, other studies presented data that corroborate this proposition. In the study by Wong et al. [10] the use of a specific instrument revealed several sexual problems that had not been detected. Similarly, in a research with Italian women, the use of a specific instrument doubled the prevalence of sexual problems in comparison to self-report [48]. Thus, indicating the difference in the diagnostic ability of simple reporting compared to the use of an appropriate specific instrument. From the results of the present study and other related research, the use of instruments/questionnaires for sexual health assessment is therefore recommended. Clegg, Towner and Wylie [49] recommend the use of questionnaires as part of the overall assessment in routine women’s health care, but without replacing the medical history.

A thorough and detailed anamnesis is the basis for all sexual health assessment, so specific instruments should act as complements. In this sense, screening instruments present benefits in routine health care, being a simple and fast method to identify potential issues. In clinical practice, these tools can be part of routine care, making it possible to access sexual health problems even in patients who come to the health service with other demands [35]. According to Kingsberg and Simon [50] simple screening tools can help professionals, even those inexperienced in the field, to identify sexual problems and the need for further evaluation and treatment. Thus, easy-to-use and efficient screening methods for sexual problems are needed [32]. The development of these standardized and validated tools and the analysis of their benefits for clinical practice is a demand from the field [49]. . In the present study, reinforcing the aforementioned data, 24% of ob/gyns providers reported that the lack of adequate tools is a barrier to addressing sexual health in the context of care for women in the menopausal stages.

Easy-to-use and quickly applied screening instruments can also even solve the main barrier pointed out by professionals: time limitation in care to address these issues. This barrier has been reported in the literature of the area [31] as in the study by Bungener et al. [43] in which 22.7% of the professionals reported not discussing the topic due to lack of time, and Rabathaly and Chattu [23] where the limited time to discuss sexual health issues was pointed out by 59% of the professionals evaluated. Another barrier indicated, also related to the health care environment, was limited privacy in clinical care. Thus, Sobecki et al. [4] conclude that the improvement in ob/gyns practices regarding the approach to sexual health also depends on an environment that facilitates the doctor-patient relationship. In this sense, they emphasize the importance of a structure in health institutions that enables and encourages dialogue and accessibility to the health demands of the population.

A comprehensive gynecological assessment is essential for women in the perimenopause and postmenopause stages to identify and address prevalent health issues, including sexual symptoms and vaginal bleeding. These issues are the primary reasons for gynecological consultations during this phase [51] and are particularly significant in low- to middle-income countries [52]. Therefore, an effective response to the health burden of middle age is to implement and expand screening programs for these conditions, given the multimorbidity of middle-aged women and its impact on quality of life [53].

An analysis of the study’s strengths and limitations looked at factors associated with participation in web surveys [54]. Considering the sampling and data collection method, it is possible that interest in the research topic influenced the choice to participate. In addition, a higher rate of participation by women in websurveys has been observed [54], a characteristic that occurred in this study.The gender, age, and geographic distribution profile of the sample was consistent with the profession’s profile in Brazil, since the average age of Brazilian physicians is 45 years; women are the majority among ob/gyns specialists; and SP is the Brazilian state with the most specialists in the area [55]. However, due to the nature of the study, the results cannot be generalized to other groups different from the specific clipping that was evaluated. Due to the limitations of this study, it is recommended the reproduction of this investigation in other population contexts and the expansion of the sample and of the questions analyzed, also including possible differences in sexual health care between the different menopause-related phases and depending on social markers of difference such as race and class of the service users. It is also interesting to extend the stratification of professionals surveyed, for example, to identify differences by type of workplace (rural or urban). Moreover, in terms of methodological limitations of the study, we emphasize the restrictions imposed by the use of a questionnaire for data collection not yet validated. In this sense, the validation of specific instruments is recommended, aiming at expanding and standardizing the data, as well as refining the comparative analyses between populations.

## Conclusions

The study revealed discrepancies in the frequency of addressing sexual health topics in healthcare for menopausal women in Brazil. Time limitations and patients’ discomfort with the subject were identified as the main barriers to addressing the topic. The use of specific tools and an active approach had a significant impact on the frequency of addressing sexual health issues.

Therefore, screening tools that address multiple aspects of sexual health in menopausal women may be beneficial in routine health care, helping to resolve discrepancies found between related topics and overcoming the main barriers reported by professionals. These strategies can effectively address the underreporting of sexual complaints among menopausal women and expand access to appropriate sexual health care for the population, while helping to manage the health burden of midlife.

### Electronic supplementary material

Below is the link to the electronic supplementary material.

**Supplementary Material 1:** Data collection questionnaire developed by the study team.

## Data Availability

The dataset generated and analysed during the current study is not publicly available due to promises of participant anonymity and confidentiality but is available from the corresponding author upon reasonable request.

## References

[1] Cuerva MJ, Gonzalez D, Canals M, Otero B, Espinosa JA, Molero F (2018). The sexual health approach in postmenopause: the five-minutes study. Maturitas.

[2] Heiden-Rootes KM, Salas J, Gebauer S, Witthaus M, Scherrer J, McDaniel K (2017). Sexual dysfunction in primary care: an exploratory descriptive analysis of medical record diagnoses. J Sex Med.

[3] Brazil. Saúde sexual e saúde reprodutiva. Cadernos de atenção básica, 2013.

[4] Sobecki JN, Curlin FA, Rasinski KA, Lindau ST (2012). What we don’t talk about when we don’t talk about Sex1: results of a National Survey of US Obstetrician/Gynecologists. J Sex Med.

[5] Heidari M, Ghodusi M, Rezaei P, Abyaneh SK, Sureshjani EH, Sheikhi RA (2019). Sexual function and factors affecting menopause: a systematic review. J Menopausal Med.

[6] Morton L (2017). Sexuality in the older adult. Prim Care.

[7] Evcili F, Demirel G (2018). Patient’s sexual health and nursing: a neglected area. Int J Caring Sci.

[8] Bdair IA, Maribbay GL (2020). Perceived knowledge, practices, attitudes and beliefs of Jordanian nurses toward sexual health assessment of patients with coronary artery diseases. Sex Disabil.

[9] Fennell R, Grant B (2019). Discussing sexuality in health care: a systematic review. J Clin Nurs.

[10] Wong EL, Huang F, Cheung AW, Wong CK (2018). The impact of menopause on the sexual health of Chinese cantonese women: a mixed methods study. J Adv Nurs.

[11] Abdo CHN, Oliveira WM, Moreira ED, Fittipaldi JA (2002). Sexual profile of Brazilian population: results from Brazilian study of sexual behavior (BSSB). Revista Brasileira De Medicina.

[12] Polland AR, Davis M, Zeymo A, Iglesia CB (2019). Association between comorbidities and female sexual dysfunction: findings from the third National Survey of sexual attitudes and lifestyles (Natsal-3). Int Urogynecol J.

[13] Abdo CHN. Female sexual quotient: a Brazilian questionnaire to assess women’s sexual activity. Diagn Tratamento 2009:89–90.

[14] Lara LA, Scalco SCP, Rufino AC, de Paula SRC, Fernandes ES, de Lima Pereira JM (2021). Management of hypoactive sexual desire disorder in women in the gynecological setting. Revista Brasileira De Ginecologia E Obstetrícia/RBGO Gynecology and Obstetrics.

[15] Lara LA, da Scalco S, Troncon SCP, Lopes JK (2017). A model for the management of female sexual dysfunctions. Rev Bras Ginecol Obstet.

[16] Rodrigues Júnior OM, Costa M. The Brazilian gynecologist and human sexuality: II. The approach to the patient’s sexuality. Reproduçäo 1987:235–9.

[17] Vieira TCB, de Souza E, Nakamura MU, Mattar R (2012). Sexuality during pregnancy: are Brazilian doctors prepared to deal with these issues?. Revista Brasileira De Ginecologia E Obstetrícia.

[18] Vieira TCSB, de Souza E, da Silva I, Torloni MR, Ribeiro MC, Nakamura MU (2015). Dealing with female sexuality: training, attitude, and practice of obstetrics and gynecology residents from a developing country. J Sex Med.

[19] Abdo CHN, Fleury HJ (2006). Diagnostic and therapeutic aspects of female sexual dysfunctions. Archives of Clinical Psychiatry.

[20] Brazil. 2010 Demographic Census. Instituto Brasileiro de Geografia e Estatística 2010.

[21] Brazil. Technical note - on the composition of the per capita household income variable used in the construction and analysis of income distribution in the continuous PNAD 2019. Instituto Brasileiro de Geografia e Estatística; 2019.

[22] Alexandre NMC, Coluci MZO (2011). Content validity in the development and adaptation processes of measurement instruments. Ciênc saúde Coletiva.

[23] Rabathaly PA, Chattu VK (2019). Sexual healthcare knowledge, attitudes, and practices among primary care physicians in Trinidad and Tobago. J Family Med Prim Care.

[24] Nusbaum MR, Gamble G, Skinner B, Heiman J (2000). The high prevalence of sexual concerns among women seeking routine gynecological care. J Fam Pract.

[25] Conselho Federal de Medicina. RESOLUÇÃO CFM N^o^ 2.221/2018. 2018.

[26] Febrasgo. Gynecology and Obstetrics Competency Matrix. Federação Brasileira das Associações de Ginecologia e Obstetrícia 2019.

[27] Nayak S, Binil V, Christabel S. Depressive Symptoms and Bio-psychosocial Problems among Postmenopausal Women of Udupi District, Karnataka, India. JCDR. 2019. 10.7860/JCDR/2019/38164.12455.

[28] Parish SJ, Clayton AH (2007). Continuing medical education: sexual medicine education: review and commentary (CME). J Sex Med.

[29] Urbina AAS, Soto ECJ (2013). The confrontation of sexuality in the professional practice of future physicians: the viewpoint of medical interns. Ciênc saúde Coletiva.

[30] Avasthi A, Grover S, Sathyanarayana Rao TS (2017). Clinical practice guidelines for management of sexual dysfunction. Indian J Psychiatry.

[31] American College of Obstetricians and Gynecologists’ Committee on Practice Bulletins—Gynecology (2019). Female sexual dysfunction: ACOG Practice Bulletin Clinical Management guidelines for Obstetrician-Gynecologists, Number 213. Obstet Gynecol.

[32] Thomas HN, Neal-Perry GS, Hess R (2018). Female sexual function at midlife and beyond. Obstet Gynecol Clin.

[33] Araujo MAL, Uesono J, Machado NM da, Pinto S, Amaral VM. E. Brazilian protocol for sexually transmitted infections 2020: approaching sexually active individuals. Rev Soc Bras Med Trop 2021;54. 10.1590/0037-8682-628-2020. 10.1590/0037-8682-628-2020 PMC8210482 34008727

[34] Ferreira B, de O, Bonan C, Ciência (2020). & Saúde Coletiva.

[35] Hatzichristou D, Kirana P-S, Banner L, Althof SE, Lonnee-Hoffmann RAM, Dennerstein L (2016). Diagnosing sexual dysfunction in men and women: sexual history taking and the role of Symptom scales and questionnaires. J Sex Med.

[36] Hegde D, Sreedaran P, Pradeep J (2018). Challenges in taking sexual history: a qualitative study of Indian postgraduate psychiatry trainees. Indian J Psychol Med.

[37] Brazil. National health survey 2019: life cycles. Instituto Brasileiro de Geografia e Estatística; 2021.

[38] Rizvi SJ, Yeung NW, Kennedy SH (2011). Instruments to measure sexual dysfunction in community and psychiatric populations. J Psychosom Res.

[39] Savoy M, O’Gurek D, Brown-James A (2020). Sex Health History: Techniques Tips Am Fam Physician.

[40] Zéler A, Troadec C (2020). Doctors talking about sexuality: what are the patients’ feelings?. Sex Med.

[41] Seitz T, Ucsnik L, Kottmel A, Bitzer J, Teleky B, Löffler-Stastka H (2020). Let us integrate sexual health—do psychiatrists integrate sexual health in patient management?. Archives of Women’s Mental Health.

[42] Gleser H (2015). Sex, women and the menopause: are specialist trainee doctors up for it? A survey of views and attitudes of specialist trainee doctors in Community Sexual & Reproductive Health and Obstetrics & Gynaecology around sexuality and sexual healthcare in the (peri)menopause. Post Reprod Health.

[43] Bungener SL, Post L, Berends I, Steensma TD, de Vries ALC, Popma A (2022). Talking about sexuality with Youth: a Taboo in Psychiatry?. J Sex Med.

[44] Rezende FCB, Lisboa HKS, Almeida LAV, Lima ER, Souza MS, Barbosa RAA et al. A sexualidade da mulher no climatério. Revista Da Universidade Vale Do Rio Verde 2019;17.

[45] Rocha AW, Cosme do Nascimento EG, Pessoa Junior JM, Carlos Alchieri J. As incertezas de mulheres em vivenciar a sexualidade no climatério. J Nurs UFPE/Revista De Enfermagem UFPE 2014;8.

[46] Brazil. Sexual rights, reproductive rights and contraceptive methods. Ministério da Saúde; 2009.

[47] Yanikkerem E, Göker A, Çakır Ö, Esmeray N (2018). Effects of physical and depressive symptoms on the sexual life of Turkish women in the climacteric period. Climacteric.

[48] Cagnacci A, Venier M, Xholli A, Paglietti C, Caruso S (2020). Female sexuality and vaginal health across the menopausal age. Menopause.

[49] Clegg M, Towner A, Wylie K (2012). Should questionnaires of female sexual dysfunction be used in routine clinical. Practice? Maturitas.

[50] Kingsberg SA, Simon JA (2020). Female hypoactive sexual Desire disorder: a practical guide to causes, clinical diagnosis, and treatment. J Women’s Health.

[51] Ulin M, Ali M, Chaudhry ZT, Al-Hendy A, Yang Q (2020). Uterine fibroids in menopause and perimenopause. Menopause.

[52] Nguyen PN, Nguyen VT (2022). Assessment of paraclinical characteristics in peri- and postmenopausal bleeding women: is there a correlation between hemoglobin levels and ultrasonic indices?. J Taibah Univ Med Sci.

[53] Puri P, Sinha A, Mahapatra P, Pati S (2022). Multimorbidity among midlife women in India: well-being beyond reproductive age. BMC Womens Health.

[54] Keusch F (2015). Why do people participate in web surveys? Applying survey participation theory to internet survey data collection. Manage Rev Q.

[55] Scheffer M, Alex Cassenote AGA, Guilloux A, Biancarelli. Giulia Marcelino Mainardi, Bruno Alonso Miotto. São Paulo: Departamento de Medicina Preventiva da Faculdade de Medicina da USP; Conselho Regional de Medicina do Estado de São Paulo; Conselho Federal de Medicina; 2018. Demografia Médica no Brasil 2018.

