# Supplementary material for: Knowledge, practices and barriers to access sexual health of women in the menopausal stages: a cross-sectional study with Brazilian gynecologists
Source: BMC Womens Health. 2024 Jan 18;24:52. doi: 10.1186/s12905-024-02901-x (PMC10795360; doi:10.1186/s12905-024-02901-x)
Supplement: Supplementary file 1 — Supplementary Material 1: Data collection questionnaire developed by the study team. [file 12905_2024_2901_MOESM1_ESM.pdf]

KNOWLEDGE, PRACTICES AND BARRIERS TO ACCESS SEXUAL HEALTH OF WOMEN IN THE MENOPAUSAL STAGES

Data Collection Questionnaire

- **SOCIOECONOMIC AND PROFESSIONAL CHARACTERISTICS**

Biological Sex
( ) Female
( ) Male

Gender Identity
____

How do you classify your color or race?
( ) White
( ) Black
( ) Yellow
( ) Brown
( ) Indigenous

Age (in years)
____________ (answer in numbers)

What is your estimated household income per person? (Sum of the income received by each resident, divided by the total number of residents in the household)
____________ (answer in numbers)

Do you have a medical degree recognized in Brazil?
( ) Yes
( ) No

How long (in years) have you been trained in medicine? (If between 1 and 11 months, check 1)
____________ (answer in numbers)

Do you have a specialization in Gynecology and Obstetrics recognized in Brazil?
( ) Yes
( ) No

How long (in years) have you been a specialist in Gynecology and Obstetrics? (If between 1 and 11 months, check 1)
____________ (answer in numbers)

Do you have any training and/or other specialization in Sexuality?
( ) Yes
( ) No

Do you work in health care with women in the menopausal transition, climacteric and/or post-menopause?
( ) Yes
( ) No

Predominant age among the patients you care for (age profile you usually work with)
( ) Less than 30 years old
( ) 30-39 years old
( ) Aged 40-49
( ) Aged 50-59
( ) Aged 60 and over

Place of Work:
(List with options - states of Brazil in alphabetical order)

Health Care System of Work (more than one option can be ticked)
( ) Private Health System
( ) Public Health System - Municipal Level
( ) Public Health System - State Level
( ) Public Health System - Federal Level

Sphere of Activity (more than one option can be ticked)
( ) Primary Care
( ) Secondary Care
( ) Tertiary and Hospital Care

In the context of the COVID-19 pandemic, how are gynecological consultations with women in the climacteric currently being carried out?
( ) Exclusively in person
( ) Exclusively remote
( ) In person and remote

- **KNOWLEDGE**

The following items refer to your knowledge of the approach to Sexuality in Health Care for Climacteric Women.

I am qualified in terms of technical and theoretical knowledge, to address Sexuality in Health Care
( ) Totally Agree
( ) Partially agree
( ) Neither agree nor disagree
( ) Partially disagree
( ) Totally Disagree

I am qualified, in terms of technical and theoretical knowledge, to address Sexuality in Health Care with Climacteric Women
( ) Totally Agree
( ) Partially agree
( ) Neither agree nor disagree
( ) Partially disagree
( ) Totally Disagree

Sexuality is part of women's health care routine in the climacteric
( ) Totally Agree
( ) Partially agree
( ) Neither agree nor disagree
( ) Partially disagree
( ) Totally Disagree

Sexuality is part of the responsibilities/attributions of the gynecologist's professional practice
( ) Totally Agree
( ) Partially agree
( ) Neither agree nor disagree
( ) Partially disagree
( ) Totally Disagree

Sexuality is an important theme in the clinical care of climacteric women
( ) Totally Agree
( ) Partially agree
( ) Neither agree nor disagree
( ) Partially disagree
( ) Totally Disagree

- **PRACTICES**

The following questions refer to your professional practice in relation to the approach to Sexuality in Health Care for Climacteric Women.

When approaching the subject of sexuality in clinical practice with women in the climacteric, most of the time, you:
( ) Proposes the topic directly (through specific questions or speeches)
( ) Waits for the patient to show interest or demand regarding the subject
( ) This subject is not often addressed

To approach the subject of sexuality in clinical practice with women in the climacteric period, most of the time you have to..:
( ) Uses an instrument/questionnaire/interview model previously drawn up from the literature in the area.
( ) Use an instrument/questionnaire/interview model previously prepared by you or the health service team you work with.
( ) Does not use a previously prepared instrument/questionnaire/interview model ( ) This subject is not frequently discussed

How often is the subject of sexuality addressed in your clinical practice with climacteric women?
( ) Very often
( ) Often
( ) Occasionally
( ) Rarely
( ) Never

Below are some topics that may be associated with experiencing and expressing sexuality. Please take a minute to think about them and indicate how often each one is addressed in your clinical practice with climacteric women:

Sexual Activities
( ) Very often
( ) Often
( ) Occasionally
( ) Rarely
( ) Never

Sexual Orientation
( ) Very often
( ) Often
( ) Occasionally
( ) Rarely
( ) Never

Relationship with yourself
( ) Very often
( ) Often
( ) Occasionally
( ) Rarely
( ) Never

Relationship with partners
( ) Very often
( ) Often
( ) Occasionally
( ) Rarely
( ) Never

Desire
( ) Very often
( ) Often
( ) Occasionally
( ) Rarely
( ) Never

Excitement
( ) Very often
( ) Often
( ) Occasionally
( ) Rarely
( ) Never

Orgasm
( ) Very often
( ) Often
( ) Occasionally
( ) Rarely
( ) Never

Satisfaction
( ) Very often
( ) Often
( ) Occasionally
( ) Rarely
( ) Never

Vaginal lubrication
( ) Very often
( ) Often
( ) Occasionally
( ) Rarely
( ) Never

Sexual Complaints and Dysfunctions
( ) Very often
( ) Often
( ) Occasionally
( ) Rarely
( ) Never

Dyspareunia and sex-related pain
( ) Very often
( ) Often
( ) Occasionally
( ) Rarely
( ) Never

- **PERCEIVED BARRIERS**

Among the following topics, please select those that you think act as barriers to addressing sexuality in the context of health care for women in climacteric conditions:
(More than one option can be selected)

- Patient's age
- Difference in biological sex and/or gender identity between patient and professional
- Time constraints in clinical care
- Lack of privacy in the healthcare setting
- Lack of specific technical and theoretical knowledge among professionals
- Lack of specialized training in professional education or continuing education
- Lack of specific guidelines for the institution in which you work
- Lack of tools to address the issue with women in the climacteric period
- Professional's personal attitudes and beliefs
- Discomfort or embarrassment about the subject (on the part of the patient)
- Discomfort or embarrassment about the subject (on the part of the professional)
- Social norms and cultural taboos on the subject
- Service via digital interface/Telecare
- Other barriers not listed
- I don't think there are any barriers to tackling the subject in this context
